# Additive-free synthesis of poly(n-vinyl pyrrolidone) and poly(n-isopropylacrylamide) nanogels with controlled sizes

**DOI:** 10.55730/1300-0527.3545

**Published:** 2023-02-01

**Authors:** S. Duygu SÜTEKİN, Feyza KIRAÇ, Olgun GÜVEN

**Affiliations:** Department of Chemistry, Faculty of Science, Hacettepe University, Ankara, Turkey

**Keywords:** Poly(vinyl pyrrolidone), poly(N-isopropylacrylamide), nanogel, gamma irradiation, size control, effect of acetone

## Abstract

An easy method is proposed to prepare poly(vinyl pyrrolidone) (PVP) and poly(N-isopropylacrylamide) (PNiPAAm) nanogels with sizes less than 100 nm. The underlying principle is to prepare dilute polymer solutions in acetone/water mixtures where acetone acts to break tridimensional structure of water hence disrupting the H-bonds bridging polymer coils causing separation and shrinkage in their sizes. Irradiation of these solutions by gamma-rays directly leads to the formation of intramolecular crosslinks within the coils resulting with nanogels with sizes smaller than precursor coils. While the average size of nanogels of PVP irradiated in water only is 236 nm, they were reduced to about 44 nm when irradiation was carried out in acetone/water solutions at near theta compositions. PNiPAAm nanogels were also synthesized by irradiating their dilute acetone/water solutions. Multimodal coil size distribution of PNiPAAm was converted into monomodal distribution with 70 nm average size and low dispersity by the addition of acetone. Irradiation of such solutions yielded PNiPAAm nanogels with 50 nm average size. Stability of nanogels was followed for 1 year not showing any changes in their sizes or size distributions. Nanogels were characterized by dynamic light scattering, scanning electron microscopy, and atomic force microscopy techniques.

## 1. Introduction

Nanogels are chemically or physically intramolecularly crosslinked polymer coils with sizes typically ranging from 10 to a few hundreds of nanometers. The crosslinked structures provide size and shape stability high water content desirable mechanical properties and high-loading capacity with additional advantages in physicochemical and rheological properties [1–3]. These unique properties make nanogels suitable for various applications, such as chemical and biological sensors [1, 2], controlled release and drug delivery systems [3, 4], contrast agents [5], biomedical implants [6], and in tissue engineering [7], and cancer treatment [8].

Starting from the first work of Staudinger when they prepared divinylbenzene microgels [9], there have been many reports on nanogels prepared from natural polymers such as chitosan [10], alginate [11], and synthetic polymers such as poly(vinyl alcohol) (PVA) [12], poly(acrylic acid) (PAA) [13], poly(vinyl pyrrolidone) (PVP) [14], poly(ethylene oxide) (PEO) [15], and poly(N-isopropylacrylamide) (PNiPAAm) [16]. In this work PVP and PNiPAAm were selected for the preparation of nanogels. PVP was preferred due to its low cytotoxicity, excellent biocompatibility with living tissue, and noncarcinogenic and nonallergic properties [17, 18]. PVP has been widely used in many biomedical applications such as drug delivery [19–21] and tissue engineering [22]. PNiPAAm was selected for its temperature responsive properties where it possesses lower critical solution temperature (LCST) in water around 32 °C [23], which is close to human body temperature. Hence, PNiPAAm nanogels show high potential for a variety of applications especially in biotechnology [24], drug delivery [25], and bioseparation [26].

Nanogels are generally prepared by one of the four methods as described by Kabanov and Vinogradov [27];

- Physical assembly of interactive polymers,
- Polymerization of monomers in a homogeneous or nanoscale heterogeneous environment,
- Crosslinking of preformed polymers,
- Template-assisted nanofabrication.

Among these methods, the easiest and the most straightforward is formation of nanogels by intramolecular crosslinking of hydrophilic or amphiphilic polymers in aqueous solutions. Intramolecular covalent bonds can be formed by using some water-soluble crosslinking agents. On the other hand, crosslinking of polymers in aqueous solutions can be achieved without using any additives or crosslinking agents if the solutions are irradiated with ionizing radiation such as gamma-rays, accelerated electrons and X-rays. Since only aqueous solutions of polymers are used with no monomers, initiators, and crosslinking agents, the nanogels synthesized upon irradiation will be free from any impurities, not requiring a purification step. As-synthesized nanogels fulfill the required specifications of the Food and Drug Administration (FDA) for biomedical applications [28]. By irradiation, it is possible to initiate the crosslinking reaction at any temperature even at subambient, and control the degree of crosslinking by simply controlling the dose rate and absorbed dose.

An important and distinguishing feature of ionizing radiation is that its absorption is nonselective as long as the electron density of irradiated medium is homogeneous so that molecules are ionized according to their relative abundance in the medium. Therefore, in dilute solutions, it is mainly the water that absorbs the radiation energy with the formation of radiolysis products. Chemically the most important and abundant species produced are the hydrated electron (e_aq_^−^) and the hydroxyl radical (^•^OH). The hydrated electron (e_aq_^−^) is the most powerful reducing agent (standard reduction potential E° = −2.78 V) and the hydroxyl radical (^•^OH) is a very powerful oxidizing agent (E°(^•^OH/OH^−^) = 1.90 V in neutral solution, and E°(H^+^, ^•^OH/H_2_O) = 2.72 V in acidic solution) [29] in aqueous solutions. Considering that irradiated water contains these two oppositely behaving species, to enhance crosslinking, one should work in totally oxidizing conditions to obtain nanogels by radiation-induced crosslinking. ^•^OH should be the major species in the crosslinking reaction. A very practical and frequently used method of converting e_aq_^−^ to ^•^OH is to saturate the aqueous solution with N_2_O ([N_2_O] ~ 25 mmol dm^−3^) [30] as it is proposed in Eqs. 1 and 2:


(1) $$e_{\text{aq}}^{-}+{\text{N}}_{2}\text{O}\to {\text{N}}_{2}+{\text{O}}^{•-}$$

(2) $${\text{O}}^{•-}+{\text{H}}_{2}\text{O}⇋{\;}^{•}\text{OH}+{\text{OH}}^{-}$$

Thus, in N_2_O saturated aqueous solutions, the crosslinking reactions will be dominated. As it will be explained later instead of pure water, water/acetone mixture was used as the solvent in the preparation polymer solutions in this work. In addition to be a nonsolvent affecting the dimensions of polymer coils in their aqueous solutions, acetone is also an excellent scavenger for hydrated electrons as can be seen from Eqs. 3 and 4 [31].


(3) $$e_{\text{aq}}^{-}+{\left({\text{CH}}_{3}\right)}_{2}\text{CO}\to {\left({\text{CH}}_{3}\right)}_{2}{\overset{•}{\text{C}}\text{O}}^{-}+{\text{H}}_{2}\text{O}$$

(4) $${{\text{(CH}}_{3})}_{2}{\overset{•}{\text{C}}\text{O}}^{-}+{\text{H}}_{2}\text{O}⇋{\left({\text{CH}}_{3}\right)}_{2}\overset{•}{\text{C}}\text{OH}+{\text{OH}}^{-}$$

The reaction constant for Eq. 3 was found as 5.9 × 10^9^ M^−1^ s^−1^ [31]. After the reaction of acetone with hydrated electron, α-hydroxyalkyl radical anions are formed. These radical anions react to form alkoxy radicals depending on the pH of solution. For the radiation-induced nanogel synthesis in acetone/water mixtures, the presence of acetone is favorable since the crosslinking reactions should be carried out in oxidizing conditions where the reducing species, mainly e_aq_^−^, should be eliminated from the system.

For a given polymer, the size of its nanogels is very important since it strongly affects the circulation time in blood and thus their bioavailability in the body by evading RES (Reticulo Endothelial System) [32]. Nanogels with very small sizes can be easily removed from the body. On the other hand, particles having diameters more than 200 nm are simply filtered by spleen which will naturally decrease circulation time of the particle [33]. Moreover, it should be noted that coil size plays an important role in the responsiveness to external stimuli since the response rate towards change of stimuli is inversely proportional to the square of the size of the gel [34]. In order to control the coil size and avoid the microgelation problem, different parameters (e.g., total absorbed dose [14, 33, 35, 36], dose rate [33], polymer concentration [33, 36], and temperature [33]) have been extensively studied in the radiation-induced synthesis of nanogels. Since polymer coils are directly converted into nanogels by radiation-induced intramolecular crosslinking, being the precursors of nanogels, the coils will determine the sizes of resulting nanogels. This gave us the idea of first controlling the sizes of polymer coils in their aqueous solutions by considering polymer solution thermodynamics and then irradiating those solutions to obtain nanogels with desired sizes. By preparing the solutions of polymers in poor solvent environment, it is possible to reduce the sizes of coils by contraction of the chains. The purpose of this study is to control the particle sizes of PVP and PNiPAAm nanogels by controlling the solution thermodynamics of respective polymer solutions by using nonsolvent effect of acetone for PVP and its hydrogen bond breaking action in acetone/water mixtures.

## 2. Materials and methods

### 2.1. Materials

Poly (N-vinyl pyrrolidone) (BASF), M_w_ = (1.278 ± 0.023) × 10^6^ g mol^−1^ (determined by static light scattering) was used as received without further purification. PNiPAAm (M_n_=40000 gmol^−1^ and Đ=1.09) was synthesized [37] by radiation-induced RAFT polymerization of N-isopropylacrylamide (NiPAAm) in N,N-dimethylformamide (DMF) using Cyanomethyl Dodecyl Trithiocarbonate (CDTC) as the RAFT agent which was obtained from Sigma & Aldrich Chemical Co. Ltd. These were high purity products and used as received other than NiPAAm which was recrystallized from hexane. In nanogel synthesis, acetone was used as received from Sigma & Aldrich Chemical Co. Ltd. (>%99.7 HPLC). Water used was purified and deionized by Milli Q system.

### 2.2. Preparation of PVP and PNiPAAm nanogels

PVP and PNiPAAm nanogels were prepared via radiation-induced crosslinking of their aqueous acetone solutions. PVP was dissolved in acetone/water mixtures with acetone volume fractions of 0.60, 0.62, 0.64, and 0.66. For PNiPAAm, stock solutions were prepared in acetone/water mixtures with 4 different acetone volume fractions 0.025, 0.05, 0.075, and 0.1. The solutions were prepared freshly and divided into 5-mL aliquots in glass vials sealed with rubber septa and saturated with N_2_O for 10 min prior to irradiation. A ^60^Co source with a dose rate of 0.8 kGy/h was used for irradiation of PVP solutions at ambient temperature. PNiPAAm solutions were placed in ^60^Co source and irradiated at a dose rate of 0.26 kGy/h at room temperature. Samples were taken from the irradiation chamber at different time intervals to adjust the total absorbed dose as 5, 10, 15 kGy for PVP and 5, 10 kGy for PNiPAAm.

### 2.3. Characterization of nanogels

#### 2.3.1. Dynamic light scattering (DLS)

In order to determine the average coil size and coil size distribution (dispersity) of PVP and PNiPAAm coils and their corresponding nanogels, Zetasizer Nano ZS (Malvern Instruments Ltd., UK) equipment available in National Nanotechnology Research Center, UNAM at Bilkent University was used. The instrument uses a 4 mW He-Ne laser (633 nm wavelength) and noninvasive backscatter (NIBS) optics that allows detecting the scattering information at 173° which is known as backscatter detection. The software of the instrument gives also a width parameter known as polydispersity or polydispersity index (PDI), which is a number indicative for particle size distribution. Accuracy of the instrument was frequently checked using the standard sample provided by Malvern. Coil size and size distribution were also randomly checked by repeated measurements of the same sample. All the experiments were carried out at constant temperature, 25 °C. Dispersants’ refractive indices and viscosity determinations, which were necessary for the calculation of intensity-based size and size distributions, were measured with an Abbe refractometer and a cone and plate viscometer (Brookfield) respectively.

#### 2.3.2. Scanning electron microscopy (SEM)

For the scanning electron microscopy analysis, the nanogel solutions were cast on silicone surface and the samples were sputter-coated before imaging, using a precision etching coating system (PECS, 682, Gatan Inc, Pleasanton, CA) with 8 nm thick gold/palladium. The size and shape of the nanogels were investigated using a scanning electron microscope (ESEM, FEI Quanta 200 FEG, FEI Company). The analyses were made in high vacuum and at relatively low acceleration voltage (5 kV) using backscattered electron technique.

#### 2.3.3. Atomic force microscopy (AFM)

Veeco Multimode™ V scanning probe microscope (Veeco Metrology LLC, Santa Barbara, CA) with Nanoscope^®^ IV controller was used to obtain AFM images of nanogels. The analysis was performed with 1–10 ohm-cm phosphorus (n) doped Si tips (Veeco, MPP-11100-140) with f_0_ values of 70–92 kHz in tapping mode and at room temperature. Force modulation probe is used which is more useful for detecting soft and stiff areas on substrates which exhibit overall uniform topography. Samples were prepared by casting 20-μL solutions on mica surface and allowing drying at room temperature before analysis. Several regions of the sample have been scanned for a reliable interpretation.

## 3. Results and discussion

When dilute aqueous polymer solutions are exposed to ionizing radiation, carbon-centered free radicals generated directly or indirectly on polymer chains are consumed by recombination, disproportionation, and hydrogen transfer or scission reactions. Among these, the radical recombination reactions within the polymer coils leads to the formation of nanogels. The reaction parameters should therefore be optimized for the recombination type of reactions to dominate the others at the same time avoiding the polymers to undergo microgelation by intermolecular crosslinking. These conditions are fulfilled when the polymer chains are well separated from each other and when high numbers of radicals are generated on a single chain [35]. The first requirement can be achieved by working in very low polymer concentrations and the latter by high dose rate irradiations.

### 3.1. Size control of PVP nanogels

The promoting factors to control the intramolecular combination of chains within a coil, hence the size of nanogel, can be widened by considering a different and versatile thermodynamic approach. Since the starting material’s (precursor PVP) coil size imposes a limitation on the nanogel size, solvent that would directly affect size of the coil will be of importance. As it is well known from polymer solution thermodynamics, the better the solvent, the more expanded will be the coils and vice versa. Therefore, solvent/nonsolvent pairs can be used to control the sizes of polymer coils. The extreme case of contraction of coils is observed under so-called theta conditions. Thus, approaching theta conditions will be an easy and practical tool in reducing the polymer coil sizes in dilute solutions for radiation-induced synthesis of nanogels since polymer coils are precursors of nanogels. In the following paragraphs, we shall demonstrate the use of such an approach in controlling the coils sizes of PVP in aqueous solutions and consequently the sizes of nanogels obtained from them.

**Figure 1:**
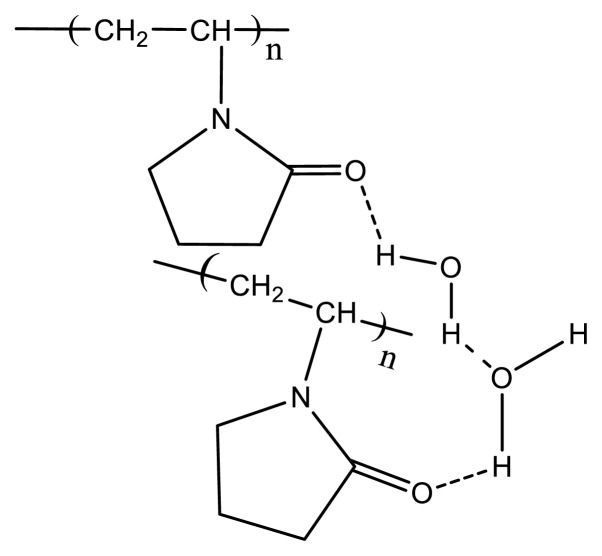
Association of PVP chains in aqueous solutions.

PVP is a hydrophilic, highly water-soluble polymer. The highly electronegative oxygen atom on the pyrrolidone ring provides a very good site for hydrogen bonding. Dimers, trimers, or oligomers of water molecules hydrogen bonded to a PVP chain may act as a bridge connecting PVP chains together. It is known that water molecules bound by PVP has a structure very close to that of ice. An ice-like structure of water attached to PVP chains confirms the view that the extent of hydrogen bonding is quite high [37]. Thus, we may assume that in aqueous PVP solutions, PVP chains are in association with each other through water molecules, hydrogen bonds being responsive for this linkage (Figure 1). Therefore, breaking of these hydrogen bonds or lowering the polymer-solvent interactions in a controlled way will result in the collapse of a pseudonetwork structure displayed by this polymer in aqueous solutions. This can also be achieved by increasing the temperature of PVP aqueous solutions [33, 38] or by using denaturing agents [37].

An extensive work on flexibility and hydrodynamic properties of PVP has been done by Abdelazim et al. by determining the unperturbed dimensions of PVP by viscosity measurements in cosolvent systems [39]. They investigated the effect of water/acetone binary mixtures to determine the theta condition for PVP and found that the theta composition of water/acetone for PVP at room temperature is Φ (acetone) = 0.668 which is in good agreement with other studies [40],[41].

**Figure 2:**
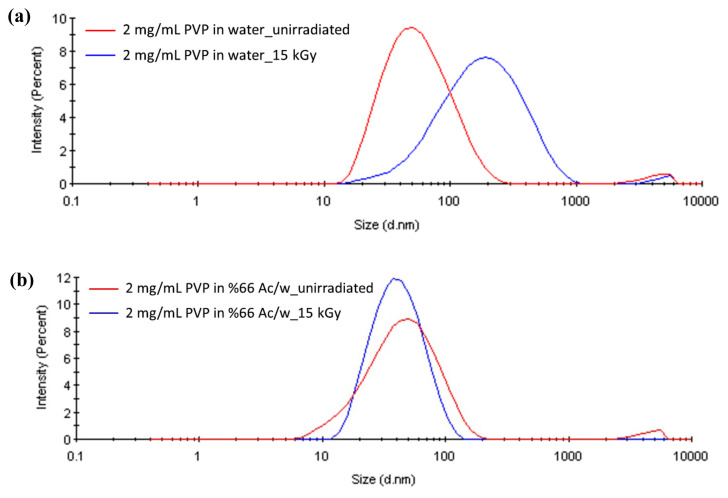
Size distribution curves based on scattered light intensities of (a) unirradiated PVP and PVP nanogels prepared in water, (b) unirradiated PVP and PVP nanogels prepared in 0.66 (v/v) acetone/water mixture from 2 mg/mL PVP solutions using gamma rays with a total absorbed dose of 15 kGy.

**Figure 3:**
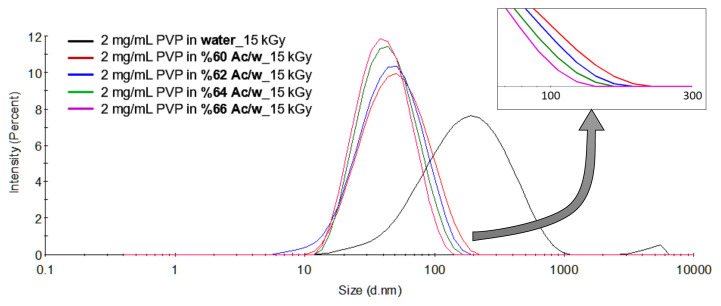
Size distribution of pristine PVP and PVP nanogels prepared in acetone/water mixtures with acetone ratios between 0.60 and 0.66 from 2 mg/mL PVP solutions using gamma rays with a total absorbed dose of 15 kGy.

Based on the information given in the preceding paragraph, we have used acetone to control the size of PVP coils in aqueous solution. Dynamic light scattering study of aqueous PVP solution revealed the fact that sizes of PVP coils cover a range of 10–120 nm as seen from Figure 2a. This is consistent with an average Rg value of 53.3 ± 1.7 nm as determined from a Zimm plot constructed by static light scattering not shown here. The nanogels formed by irradiation of this solution to 15 kGy, however, cover a size range of 10–1000 nm. Although PVP concentration of 2 mg/mL used here is below the critical concentration of 3.4 mg/mL for this particular molecular weight, it is obviously seen that there is a significant amount of crosslinking taking place intermolecularly resulting with nanogels of much larger sizes of around 236 nm with very large dispersity. Neither the large size nor high dispersity of these nanogels is apt for their utilization as nanocarriers for biomedical applications. Their sizes should be reduced and dispersity should be made as narrow as possible. When 2 mg/mL PVP solution is prepared in acetone/water solvent with 0.66 acetone volume fraction DLS curve shown in Figure 2b was observed to shift to lower sizes as compared to acetone absent distribution. As seen in Figure 2b, 15 kGy irradiation of the latter yielded smaller size nanogels with narrow size distribution. In pure water, the H-bonded network is predominant and high density of H-bonds forming a tridimensional network is responsible for the association of PVP chains. When acetone is added to such a system, the network becomes progressively disordered because acetone can form H-bonds with water. H-bonds formed between “C=O” group of acetone and the “OH” group of water has been well studied and proven by an intensive FTIR investigation [42]. Disruption of the chains of hydrogen bonds by the presence of acetone will loosen the PVP-PVP interactions and by causing the creation of more hydrophobic environment around PVP chains with consequent contraction of chains, smaller coils. Nanogels to be obtained from these smallish coils will acquire even smaller sizes. The effect of concentration of acetone on the nanogels can be seen from the respective size distribution curves shown in Figure 2. It was observed that PVP coils underwent a clear shrinkage in size in the presence of acetone where shrinkage from 63 nm (water) to 51 nm (%66 Ac/w) was observed with the introduction of acetone into the system. The sizes of nanogels obtained from these solutions were 56 and 44 nm, respectively. The effect of acetone concentration in the vicinity of theta concentration of 0.668 acetone volume fraction can be seen in Figure 3. The size decrease in the upper end of distribution curves was clearly seen in Figure 3.

Table shows the peak mean diameters, their standard deviations, and PDI values for nanogels synthesized from 2 mg/mL aqueous PVP solutions by gamma irradiation at different acetone compositions for total absorbed doses of 5, 10, and 15 kGy. It can be clearly seen that the total absorbed dose in the range given here does not affect the size or size distribution of PVP nanogels considerably. Moreover, the PDI values in Table show that the size distributions were narrower for PVP nanogels prepared in acetone/water mixtures (0.20–0.23) than those prepared in water only (0.31–0.38).

**Figure 4:**
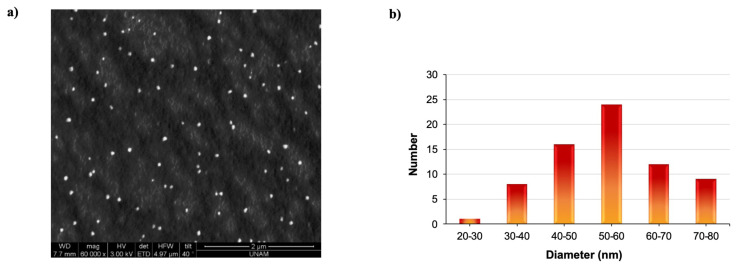
a) Scanning electron micrograph of PVP nanogels prepared from 2 mg/mL solution by gamma irradiation with a total absorbed dose of 15 kGy, magnification 60,000×, b) histogram of size distribution of PVP nanogels as determined by SEM analysis.

The synthesized PVP nanogels were characterized by scanning electron microscopy. Figure 4a shows SEM images of PVP nanogels prepared in 0.66 acetone/water mixture from 2 mg/mL solution of PVP using gamma rays with a total absorbed dose of 15 kGy, with a magnification of 60,000×. It is seen that the particles are mostly spherical in shape and homogeneous in size. The majority of particle sizes were found to be between 40 and 50 nm as obtained from the histogram generated from SEM image of PVP nanogels (Figure 4b), which was as expected due to the shrinkage of nanogels in their dried state.

**Figure 5:**
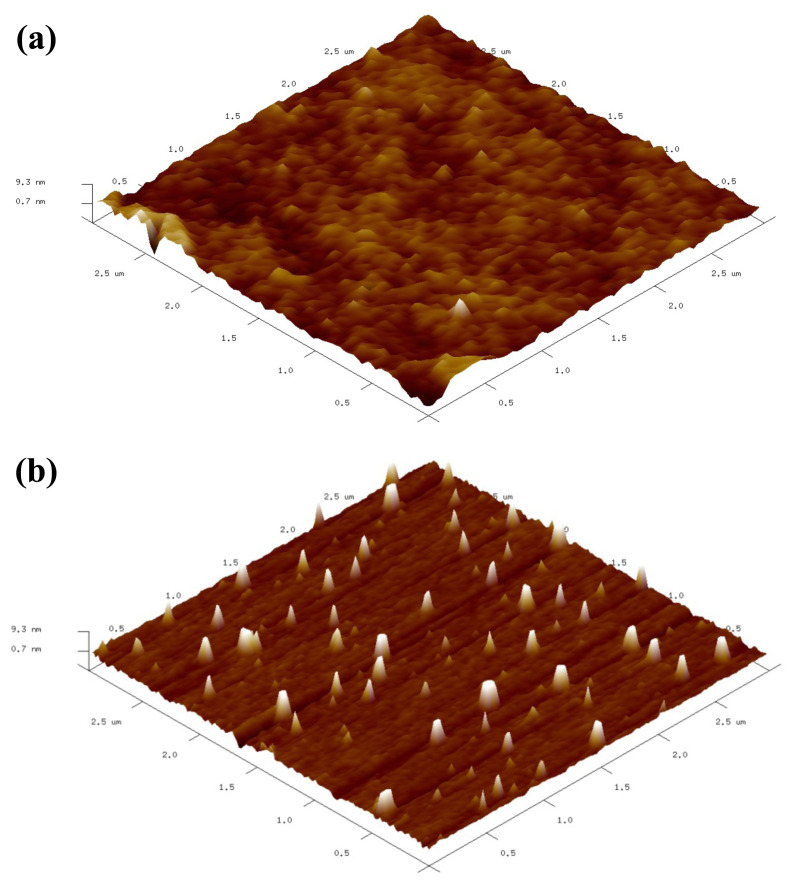
a) 3D views of AFM images of PVP coated mica surface, b) PVP nanogels deposited on mica surface.

Atomic force microscopy analysis was also performed to investigate the sizes and size distributions of PVP nanogels topographically. Figure 5 shows the 3D views of the AFM images of PVP-coated mica surface (A) and PVP nanogels deposited mica surface (B). Mica surface was chosen as the support material since it has very homogeneous profile with very small roughness. After the deposition of soluble PVP, linear PVP chains coated the surface as a flat film (Figure 5a). When PVP nanogel solution was dried on mica surface, the small spikes arising from the deposited nanogels are clearly visible (Figure 5b). Particle size analysis was performed using the software of the instrument and the average size of nanogels was found to be 42 nm.

Once the important role played by nanogels in biomedical applications was understood, scientists sought for methods to synthesize them with controlled sizes and affinities. It has been very well documented that the optimum size of nanogels are around 70 nm for maximum bioavailability [33].

For the synthesis of PVP nanogels, alternative routes have been proposed. Bueno et al. synthesized PVP nanogels using emulsion polymerization in reverse micelles using the Fenton reaction. They used isooctane/n-hexanol mixture as solvent and hexadecyltrimethylammoniumbromide as surfactant to prepare nanogels by using Fe^2+^ and H_2_O_2_. Using this method, they were able to obtain nanogels with 30 nm size [43]. Bharali et al. also synthesized poly(vinyl pyrrolidone) nanogel crosslinked with *N*,*N*′-methylene bis-acrylamide in the aqueous cores of reverse micellar droplets and obtained nanogels smaller than 100 nm. They used sodium bis-2-ethylhexylsulfosuccinate as surfactant, ammonium persulfate as initiator and n-hexane as the solvent [44]. However, the presence of crosslinker, surfactant and initiator and the use of organic solvents need further removal of these substances which is substantial for biomedical applications. Al-Sheikhly and coworkers made use of shrinking behavior of PVP in aqueous solutions at elevated temperatures to prepare PVP nanogels by electron beam irradiation [45]. In this study, we used gamma-induced intramolecular crosslinking method to avoid tedious purification steps and to prepare nontoxic, biocompatible PVP nanogels under mild conditions in the presence of acetone and water at room temperature. Thus, it was possible to prepare size-controlled nano-scale PVP gels by low dose gamma irradiation having particle sizes as low as 44 nm with the addition of acetone into the medium.

### 3.2. Size control of PNiPAAm nanogels

**Figure 6:**
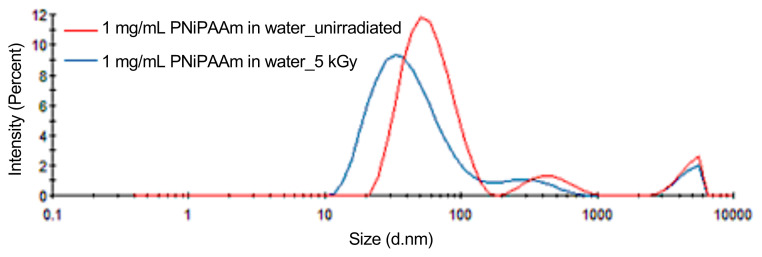
The size distributions of unirradiated and 5 kGy irradiated PNiPAAm coils (M_w_ = 40,000) determined by DLS in water.

Due to its well-understood thermoresponsive behavior, PNiPAAm has been widely used in various biomedical applications. In order to prepare its nanogels with controlled sizes by irradiation of its aqueous solutions, we planned to use a polymer sample with very low polydispersity with the anticipation of obtaining narrow coil size distribution. Thus, nanogels with small sizes and narrow distribution could possibly be obtained by simply irradiating such PNiPAAm solutions. We used a PNiPAAm sample with M_n_=40000 gmol^−1^ and Đ=1.09 that was synthesized by radiation-induced reversible addition–fragmentation chain transfer (RAFT) polymerization as described in detail in our previous publication [46]. Figure 6 shows the coil size distribution of PNiPAAm in water measured by DLS before and after gamma irradiation. Although PNiPAAm had a very narrow molecular weight distribution as determined by GPC, the coil size distribution of PNiPAAm in aqueous solution was not monomodal. The three peaks with different average sizes appear in Figure 6 corresponding to about 55, 450, and 1400 nm. The appearance of two very large sizes cannot be due to the polymer molecular weight distribution but due to the aggregation of polymer chains with these hydrodynamically favorable-sized clusters which we cannot explain. Wu and Zhou observed the presence of PNiPAAm globules with 8-fold increase in the radius of gyration for extremely dilute solution of PNiPAAm with a narrow molecular weight distribution [47]. Aseyev et al. showed that the formation of mesoglobules, colloidally stable aggregates for three different thermoresponsive polymers namely, poly(N-vinyl caprolactam), poly(N-isopropyl acrylamide), and poly(vinyl methyl ether) is a universal phenomenon with an entropic origin [48]. When these globules are formed, they neither precipitate nor disintegrate upon standing, hence not showing association among themselves. To form a stable particle in aqueous environment, the outer shell of the particle should be hydrophilic. These so-called mesoglobules of PNiPAAm would be expected to be composed of a relatively hydrophobic core surrounded by a more hydrophilic shell.

When aqueous solution of PNiPAAm was irradiated by gamma rays, the resulting nanogels showed the same trend of their polymer precursors and the original three-modal size distribution peaks maintained but shifted to lower sizes as shown in Figure 6. Therefore, it was not possible to obtain PNiPAAm nanogels having small sizes and narrow and uniform size distributions by simply irradiating aqueous PNiPAAm solutions of even very narrow molecular weight distributions by gamma rays.

**Figure 7:**
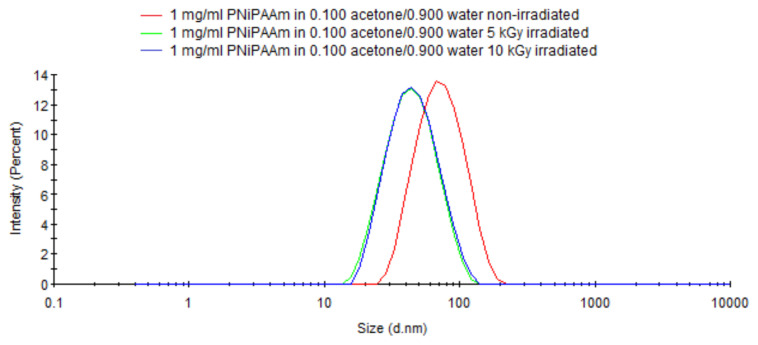
The DLS curves of PNiPAAm (40,000 MW) and its nanogels synthesized by 5 and 10 kGy gamma irradiation recorded in acetone/water (0.1/0.9 volume fraction).

Addition of acetone into aqueous PNiPAAm solution solved the problem of multimodal distribution. Figure 7 shows the DLS curve of PNiPAAm in acetone/water (0.1/0.9 v/v) as a single peak appearing at around 70 nm. Munk et al. reported that acetone and water can make three different complexes with each other. One acetone molecule can make complex with one, four, or ten water molecules [49]. By altering the tridimensional network structure of water, acetone reduces the strength of PNiPAAm-water interactions. This can be expected to destabilize large PNiPAAm aggregates with their eventual disintegration into smaller PNiPAAm coils. Thus, three-modal size distribution of PNiPAAm seen in Figure 6 is replaced by a monomodal distribution as shown in Figure 7 by the addition of acetone. Irradiation of such a solution would be expected to yield corresponding nanogels with narrow monomodal distribution. Due to the shrinkage caused by intramolecular crosslinks, the nanogels with an average size of about 50 nm were obtained from precursor coils with 70 nm size. Also seen from this figure is that increasing of dose from 5 to 10 kGy had no effect on the average size and size distribution of PNiPAAm nanogels. The sizes of PNiPAAm nanogels that were synthesized by irradiating PNiPAAm solutions in acetone/water mixtures with four different acetone volume fractions (0.025–0.10) gave nanogels with the same size, 52.5 ± 0.6 and with very low PDI = 0.171.

**Figure 8:**
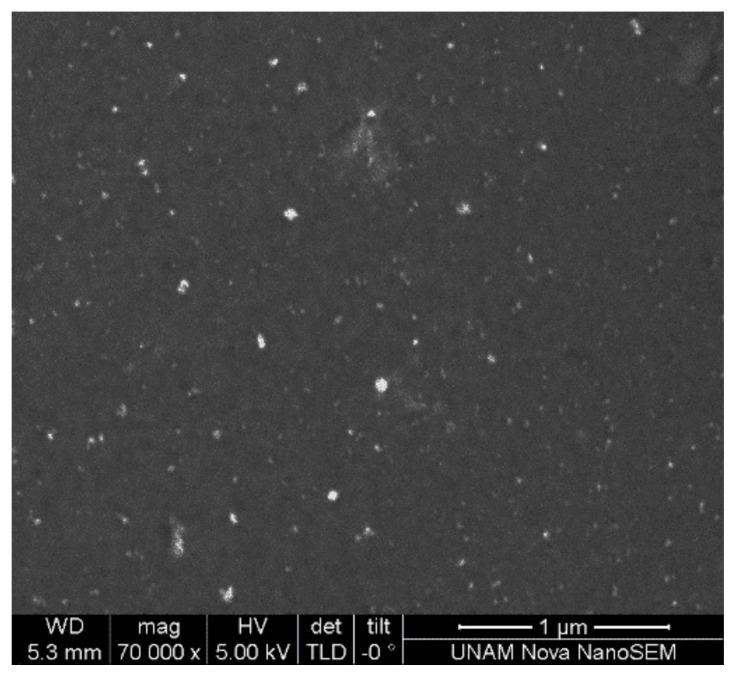
SEM image of PNiPAAm nanogels synthesized by 5 kGy irradiation in acetone/water (v/v).

**Figure 9:**
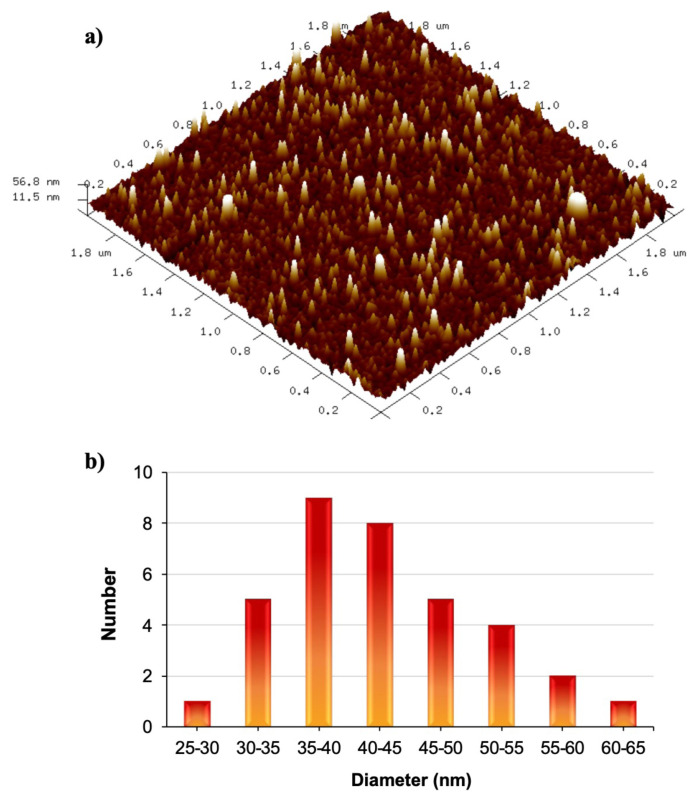
a) 3D AFM images of PNiPAAm nanogels on mica surface, b) histogram of size distribution of PNiPAAm nanogels as determined by AFM analysis.

The size of PNiPAAm nanogels was also characterized by using scanning electron microscopy and atomic force microscopy. The results obtained from these techniques were in accordance with each other. The size of dry PNiPAAm nanogels was found to be around 35 nm. In Figures 8 and 9a, SEM and 3D AFM images of PNiPAAm nanogels are given, respectively. As it was expected, the size of nanogels obtained from DLS was higher than these results because DLS measurements were done in solution. The histogram given in Figure 9b shows that particle sizes are mostly in between 35 and 40 nm due the contraction of nanogels in their dried form.

For stability studies, the size distributions of PVP and PNiPAAm nanogels synthesized by 5 kGy irradiation were analyzed by using DLS after keeping nanogels in acetone/water solution for 1 year in refrigerator. No changes were observed in either the average size of size distributions of both nanogels. The DLS curves of aged and original nanogels overlapped with each other not shown here.

The methods that have been used for the synthesis of PNiPAAm nanogels were emulsion polymerization, precipitation polymerization, and photopolymerization, where crosslinkers, surfactants, photo/chemical initiators have been used in preparation of nano- or microgels. For instance, Brijitta et al. synthesized PNiPAAm nanogels by free radical precipitation polymerization in the presence of N,N′-methylenebisacrylamide and sodiumdodecylsulfate. They measured particle size of PNiPAAm nanogels by DLS and found it as 273 nm at room temperature [50]. Borsos et al. prepared monodisperse PNiPAAm nanogel with a diameter of around 250 nm by using methylenebisacrylamide, ammonium persulfate, and dodecylbenzenesulfonic acid sodium salt at 80 °C [51]. In the study of Xiangli et al., it was also emphasized that the presence of crosslinker could cause structural inhomogeneities in nanogels; therefore, the methods which are proceeded by using crosslinker are not desirable for the synthesis of nanogels.

What we propose here, however, is a direct and clean method carried out at room temperature for the synthesis of PNiPAAm nanogels with optimum sizes for biomedical applications.

## 4. Conclusion

A simple thermodynamic approach was used to prepare PVP and PNiPAAm nanogels by controlling the sizes of precursor coils. Acetone was used to control the size and size distribution of PVP coils in water at pretheta (0.668 v/v) concentrations. The contracted coils in acetone-water mixtures were irradiated by gamma rays to induce in situ intrachain crosslinks with the formation of respective nanogels in the range of 56–44 nm. The diameters of nanogels showed no sensitivity to total absorbed dose up to 15 kGy. When 2 mg/mL PVP in water only was subjected to this dose, gels having sizes of 236 nm were obtained indicating the formation of interchain crosslinks together with intrachain crosslinks. This promising thermodynamic approach which resulted in coil shrinkage from 236 to 44 nm may be a major step forward to control the sizes of other hydrophilic polymers. Moreover, radiation-induced synthesis of PNiPAAm nanogels were also achieved in water/acetone (0.1/0.9 v/v) systems. PNiPAAm nanogels were synthesized by using 5 and 10 kGy gamma irradiation and obtained reproducibly with particle size around 50 nm whose precursor coils were around 70 nm. The size of PNiPAAm coils showed coil size reduction as a result of intramolecular crosslinking in preparation of PNiPAAm nanogels which were stable for 7 months both in solution and dry form. The PVP and PNiPAAm nanogels synthesized by gamma irradiation were analyzed by DLS, SEM, and AFM. The results obtained from all of these techniques were comparable and in accordance with each other.

## Figures and Tables

**Table:** Peak mean diameters, their standard deviations, and PDI values for nanogels synthesized from 2 mg/mL aqueous PVP solutions by gamma irradiation at the indicated doses and acetone compositions.

Gamma	Unirradiated			5 kGy			10 kGy			15 kGy		
2 mg/mL	d (nm)	std dev.	PDI	d (nm)	std dev.	PDI	d (nm)	std dev.	PDI	d (nm)	std dev.	PDI
Water	63.3	0.37	0.26	206.6	4.78	0.35	247.9	5.66	0.38	236.4	1.06	0.31
60% acetone	59.7	0.58	0.32	56.4	0.30	0.20	55.9	0.43	0.23	56.3	0.58	0.22
62% acetone	57.7	0.81	0.28	53.1	0.18	0.23	51.5	0.12	0.23	51.6	0.32	0.23
64% acetone	55.4	0.39	0.26	48.2	0.21	0.22	48.8	0.13	0.22	47.8	0.28	0.21
66% acetone	51.1	0.73	0.32	45.5	0.39	0.21	45.2	0.54	0.22	44.1	0.46	0.21
